# Auxin research: creating tools for a greener future

**DOI:** 10.1093/jxb/erad420

**Published:** 2023-12-01

**Authors:** Marta Del Bianco, Jiří Friml, Lucia Strader, Stefan Kepinski

**Affiliations:** Italian Space Agency, Rome, Italy; Institute of Science and Technology Austria (ISTA), Klosterneuburg, Austria; Department of Biology, Duke University, Durham, NC, USA; School of Biology, University of Leeds, Leeds, UK

**Keywords:** Auxin, climate change, development, plant breeding, plant immune response

**Amid the delays due to the global pandemic, in early October 2022, the auxin community gathered in the idyllic peninsula of Cavtat, Croatia. More than 170 scientists from across the world converged to discuss the latest advancements in fundamental and applied research in the field. The topics, from signalling and transport to plant architecture and response to the environment, show how auxin research must bridge from the molecular realm to macroscopic developmental responses. This is mirrored in this collection of reviews, contributed by participants of the Auxin 2022 meeting.**

The plant hormone auxin is a master regulator of plant development. Discovered more than a century ago, the organizing power of auxin lies in its transport and dynamic distribution. Central to polar auxin transport (PAT) are membrane auxin carriers, which facilitate the influx and efflux of auxin across the membrane (Han *et al*., 2021). In particular, the membrane-localized efflux carriers of the PIN (PIN-FORMED) family play a critical role in determining the direction of the auxin flow. PIN polarization in the cell membrane is very dynamic, and changes in response to various environmental and developmental cues (Friml, 2022). However, efflux carriers are not only found on the plasma membrane—members of the non-canonical PIN-FORMED and PIN-LIKES auxin transporters are localized in the endoplasmic reticulum (ER) membrane (Ung *et al.*, 2023). Mutants in the ER-localized PINs show subtle phenotypes in root architecture and pollen development. ER-localized PINs are believed to mediate auxin sequestration to the ER lumen, which is rich in auxin conjugate hydrolases. The conjugation of auxin is an important mechanism to fine-tune the plant cellular auxin levels, allowing for auxin detoxification and storage in a non-bioactive form. Regardless, whether at the plasma membrane or at the ER, PIN protein structure and the mechanism of auxin transport had remained one of the most eagerly awaited advances in the field, finally provided by an independent effort of three groups just before the meeting (Hammes *et al*., 2022).

In the cell, the pathways mediating the auxin response are complex and still not fully understood. According to the canonical nuclear auxin pathway, auxin stabilizes the interaction between the TIR1/AFB F-box proteins and the Aux/IAA transcriptional co-repressors (Del Bianco and Kepinski, 2011). This interaction prompts the degradation of Aux/IAA via the proteasome, allowing the auxin response factor (ARF) transcription factors to mediate the auxin transcriptional response. This pathway has been known for almost 20 years, but there are still many biochemical and thermodynamic details at the molecular level that are still being elucidated (Ramans-Harborough *et al.*, 2023; Wójcikowska *et al.*, 2023). This seemingly simple pathway can elicit context-specific responses thanks to the specific expression, interaction, and DNA binding abilities of the several members that constitute each protein family (Del Bianco and Kepinski, 2011; Rienstra *et al.*, 2023). MONOPTEROS (MP)/ARF5, one of the most studied ARFs, is a prime example of the versatility of the components of the nuclear auxin pathway (Wójcikowska *et al.*, 2023). MP/ARF5 regulates many aspects of plant development, including embryogenesis, meristem activity, and leaf and vasculature organogenesis, via the modulation of gene expression. By constituting a still poorly characterized protein complex, MP/ARF5 and its isoforms regulated numerous targets in an auxin concentration-dependent manner. Naturally, *mp* mutants show pleiotropic phenotypes, from severe developmental abnormalities to sterility, that vary according to the allele.

In addition to the canonical nuclear auxin pathway, an increasing body of evidence is gathering regarding new signal transduction cascades. Indeed, many responses elicited by auxin are too fast to involve a transcriptional response: changes in plasma membrane potential, cytosolic calcium spikes, apoplast acidification or alkalinization, and endomembrane trafficking. Originally, it has been generally assumed that these responses are too fast to be mediated by the TIR1/AFBs transcriptional pathway and, therefore, additional auxin perception and signalling mechanism were invoked, for lack of better candidates focusing on the ABP1 (Auxin Binding Protein1) and its interaction partner transmembrane kinase TMK1 (Fiedler and Friml, 2023). However, the important subset of these rapid responses underlying the root growth inhibition was convincingly attributed to a non-transcriptional action of TIR1/AFBs, in particular AFB1 (Fendrych *et al*., 2018; Chen *et al*., 2023; Dubey *et al*., 2023). This motivated a search for additional functionalities of TIR1/AFBs leading to an unexpected identification of adenylate cyclase (AC) activity, which seems to be an evolutionarily conserved feature of these types of receptors (Qi *et al.*, 2022). Nonetheless, the TIR1/AFB AC activity and its product, the notorious second messenger known from animals, cAMP, seems to be somehow involved in the transcriptional pathway, thus leaving the non-transcriptional branch of the TIR1/AFB pathway still elusive. On the other hand, the other rapid auxin responses related to plasma membrane ATPase activation, subcellular trafficking, and feed-back on auxin transport has now been linked to TMK1 and ABP1 (Li *et al.*, 2021), which recently emerged from one of its many controversies as an established auxin receptor for auxin canalization underlying vascular tissue formation and regeneration (Friml *et al.*, 2022). One of the main recent surprises in the field was that auxin induces an ultrafast, global phosphoresponse, which is also downstream of the ABP1–TMK perception mechanism (Friml *et al.*, 2022). It is likely that mining this rich resource of new auxin targets will reveal other physiological and developmental roles of the ABP1–TMK pathway including cell wall remodelling (Jobert *et al*., 2023).

Through its transport and response pathways, auxin regulates many aspects of plant development in a robust yet flexible way. Following the formation of the zygote, auxin acts as a special cue to guide patterning: from the embryo to inflorescence development, from meristem activity to vasculature differentiation (Möller and Weijers, 2009; Smith and Nimchuk, 2023; Sun *et al.*, 2023; Wakeman and Bennett, 2023). Extensively studied in the model plant *Arabidopsis thaliana*, recent efforts have been made to understand if and how these responses are relevant for cereal development (Cowling *et al.*, 2023; Wakeman and Bennett, 2023). Notably, cereals carry grass-specific clades of PIN transporters and auxin biosynthesis and signalling genes, with altered expression and function compared with their counterparts in Arabidopsis (Wakeman and Bennett, 2023). Grass-specific PINs are conserved in other grasses and probably involved in determining shoot architecture, although it is still unclear whether their function is linked to their structural alterations. Also in cereals, auxin crosstalks with other phytohormone inputs to regulate the immune and drought responses, root growth, shoot formation, and leaf morphogenesis (Cowling *et al.*, 2023).

The auxin system acts as a hub to integrate biotic and abiotic stress in plant growth and development. Auxin is also involved in the responses to temperature, salt stress, and drought. Salt and drought stress have been shown to act on auxin responsiveness via the post-transcriptional regulation of ARFs (Verma *et al.*, 2022). Temperature modulates cell growth by inducing auxin biosynthesis in the cotyledon, from which it is then transported to the hypocotyl and the root to trigger the appropriate developmental response (Jing *et al.*, 2023). This is accompanied by the regulation of a great number of genes, the regulation of protein stability, and the integration of different hormonal pathways via protein–protein interaction (Bianchimano *et al.*, 2023). From the point of view of biotic stress, auxin interacts with jasmonic acid (JA) and salicylic acid (SA) to initiate the plant defence response (Van der Does *et al.*, 2013). Auxin signalling has also been implicated in priming the plant defence in a phenomenon called induced systemic resistance (ISR), where the exposure to one stressor enhances the plant resistance to subsequent infections (Romera *et al.*, 2019). However, in some cases, pathogens and parasitic plants can take advantage of the auxin signalling and manipulate it to promote their own growth and infection (Kunkel and Johnson, 2021; Kirschner *et al.*, 2023). However, similar processes become beneficial while favouring positive plant–microbe interactions (Spaepen and Vanderleyden, 2011)

Understanding the complexity of auxin signalling is essential, not only to elucidate the fundamental mechanisms behind plant growth and development, but also to create tools for applications in agriculture and horticulture (Fig. 1) (Del Bianco and Kepinski, 2018). Researchers continue to study the elements of auxin transport and signalling to better understand how plants adapt to their environment and to develop new strategies for crop improvement. Indeed, the knowledge surrounding the role of auxin in plant development and response can be a valuable tool to help scientists and breeders develop crops that are more efficient, require less water, nutrient inputs, and energy, and are better equipped to withstand the challenges posed by climate change, thus ensuring the security and sustainability of food production in a rapidly changing world. However, while the potential applications of auxin research in climate-resilient crop breeding are promising, their feasibility must be carefully considered. Indeed, auxin is a powerful regulator of plant growth, so its manipulation in the context of the regulation of, for example, plant architecture needs to take into account secondary effects on the plant growth and fitness, which could affect the overall yield (Wójcikowska *et al.*, 2023). Another complex example is during plant–microbe interaction, where auxin can favour the establishment of positive associations, but can also be exploited by pathogens and parasitic plants (Spaepen and Vanderleyden, 2011; Kunkel and Johnson, 2021; Kirschner *et al.*, 2023). Central to future crop improvement efforts will be, therefore, the study of genes and pathways that fine-tune the auxin response. These genes could represent valuable targets for the creation of powerful tools for crop improvement through genetic manipulation and the identification of useful genetic variation. The study of the auxin pathway and response not only in model systems, but also in crop species, could help identify new targets though the identification of the molecular mechanisms that underlie phenotypic variation. Moreover, different local environments pose specific challenges, which will require the development of tailored crop ideotypes. Together, these efforts can create a platform for crop improvement that leverages the power of decades of fundamental auxin research with our rapidly increasing knowledge of crop genomics, genetics, and precision breeding techniques, something that will be required if we are to meet the grand challenges of the coming century.

**Fig. 1.**
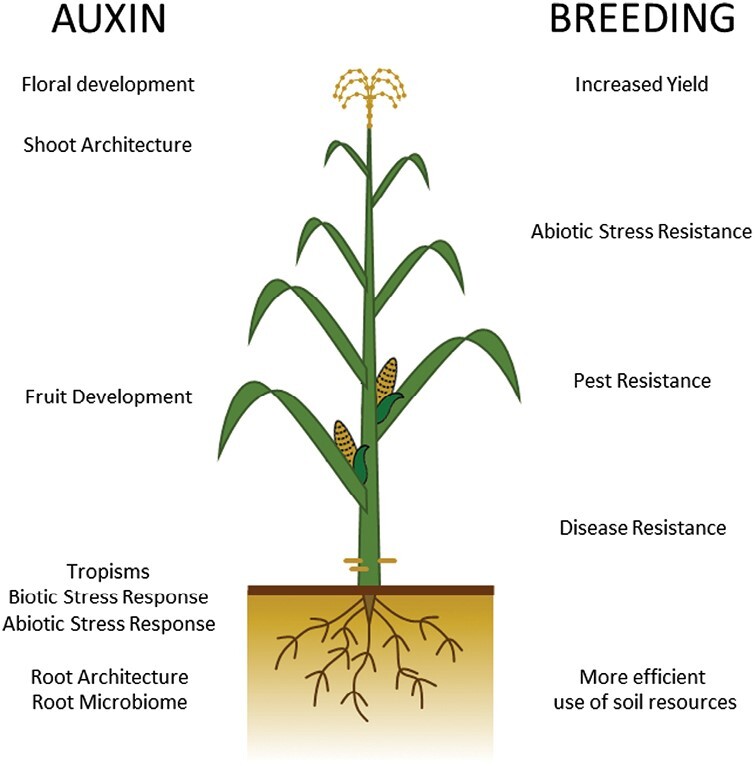
Auxin is the master regulator of plant adaptation to its environment. There is great potential for applications of auxin research in climate-resilient crop breeding; however, a holistic approach is needed to balance positive traits with possible negative effects. Central to future crop improvement efforts will be, therefore, the study of genes and pathways that fine-tune the auxin response. These genes could represent powerful tools for crop improvement through genetic manipulation. New targets could be also identified through the study of the genetic variation in the auxin pathway and response, not only in model systems, but also in crop species.

## References

[CIT0001] Bianchimano L , De Luca MB, Borniego MB, Iglesias MJ, Casal JJ. 2023. Temperature regulation of auxin-related gene expression and its implications for plant growth. Journal of Experimental Botany 74, 7015–7033. doi:10.1093/jxb/erad265 37422862

[CIT0002] Chen H , Li L, Zou M, Qi L, Friml J. 2023. Distinct functions of TIR1 and AFB1 receptors in auxin signaling. Molecular Plant 16, 1117–1119. 37393433 10.1016/j.molp.2023.06.007

[CIT0003] Cowling CL , Dash L, Kelley DR. 2023. Roles of auxin pathways in maize biology. Journal of Experimental Botany 74, 6989–6999. doi:10.1093/jxb/erad297 PMC10690729 37493143

[CIT0004] Del Bianco M , Kepinski S. 2011. Context, specificity, and self-organization in auxin response. Cold Spring Harbor Perspectives in Biology 3, a001578–a001578. 21047914 10.1101/cshperspect.a001578 PMC3003454

[CIT0005] Del Bianco M , Kepinski S. 2018. Building a future with root architecture. Journal of Experimental Botany 69, 5319–5323. 30445468 10.1093/jxb/ery390 PMC6255693

[CIT0006] Dubey SM , Han S, Stutzman N, Prigge MJ, Medvecká E, Platre MP, Busch W, Fendrych M, Estelle M. 2023. The AFB1 auxin receptor controls the cytoplasmic auxin response pathway in *Arabidopsis thaliana*. Molecular Plant 16, 1120–1130. 37391902 10.1016/j.molp.2023.06.008 PMC10720607

[CIT0007] Fendrych M , Akhmanova M, Merrin J, Glanc M, Hagihara S, Takahashi K, Uchida N, Torii KU, Friml J. 2018. Rapid and reversible root growth inhibition by TIR1 auxin signalling. Nature Plants 4, 453–459. 29942048 10.1038/s41477-018-0190-1 PMC6104345

[CIT0008] Fiedler L , Friml J. 2023. Rapid auxin signaling: unknowns old and new. Current Opinion in Plant Biology 75, 102443. 37666097 10.1016/j.pbi.2023.102443

[CIT0009] Friml J. 2022. Fourteen stations of auxin. Cold Spring Harbor Perspectives in Biology 14, a039859. 34400554 10.1101/cshperspect.a039859 PMC9159264

[CIT0010] Friml J , Gallei M, Gelová Z, et al. 2022. ABP1–TMK auxin perception for global phosphorylation and auxin canalization. Nature 609, 575–581. 36071161 10.1038/s41586-022-05187-x

[CIT0011] Hammes UZ , Murphy AS, Schwechheimer C. 2022. Auxin transporters—a biochemical view. Cold Spring Harbor Perspectives in Biology 14, a039875. 34127449 10.1101/cshperspect.a039875 PMC8805647

[CIT0012] Han H , Adamowski M, Qi L, Alotaibi SS, Friml J. 2021. PIN-mediated polar auxin transport regulations in plant tropic responses. New Phytologist 232, 510–522. 34254313 10.1111/nph.17617

[CIT0013] Jing H , Wilkinson EG, Sageman-Furnas K, Strader LC. 2023. Auxin and abiotic stress responses. Journal of Experimental Botany 74, 7000–7014. doi:10.1093/jxb/erad325 PMC10690732 37591508

[CIT0014] Jobert F , Yadav S, Robert S. 2023. Auxin as an architect of the pectin matrix. Journal of Experimental Botany 74, 6933–6949. doi:10.1093/jxb/erad174 PMC10690733 37166384

[CIT0015] Kirschner GK , Xiao TT, Jamil M, Al-Babili S, Lube V, Blilou I. 2023. A roadmap of haustorium morphogenesis in parasitic plants. Journal of Experimental Botany 74, 7034–7044. doi:10.1093/jxb/erad284 PMC10752351 37486862

[CIT0016] Kunkel BN , Johnson JMB. 2021. Auxin plays multiple roles during plant–pathogen interactions. Cold Spring Harbor Perspectives in Biology 13, a040022. 33782029 10.1101/cshperspect.a040022 PMC8411954

[CIT0017] Li L , Verstraeten I, Roosjen M, et al. 2021. Cell surface and intracellular auxin signalling for H+ fluxes in root growth. Nature 599, 273–277. 34707283 10.1038/s41586-021-04037-6 PMC7612300

[CIT0018] Möller B , Weijers D. 2009. Auxin control of embryo patterning. Cold Spring Harbor Perspectives in Biology 1, a001545. 20066117 10.1101/cshperspect.a001545 PMC2773644

[CIT0019] Qi L , Kwiatkowski M, Chen H, et al. 2022. Adenylate cyclase activity of TIR1/AFB auxin receptors in plants. Nature 611, 133–138. 36289340 10.1038/s41586-022-05369-7

[CIT0020] Ramans-Harborough S , Kalverda AP, Manfield IW, et al. 2023. Intrinsic disorder and conformational coexistence in auxin coreceptors. Proceedings of the National Academy of Sciences, USA 120, e2221286120. 10.1073/pnas.2221286120 PMC10556615 37756337

[CIT0021] Rienstra J , Hernández-García J, Weijers D. 2023. To bind or not to bind: how Auxin Response Factors select their target genes. Journal of Experimental Botany 74, 6922–6932. doi:10.1093/jxb/erad259 PMC10690724 37431145

[CIT0022] Romera FJ , García MJ, Lucena C, Martínez-Medina A, Aparicio MA, Ramos J, Alcántara E, Angulo M, Pérez-Vicente R. 2019. Induced systemic resistance (ISR) and Fe deficiency responses in dicot plants. Frontiers in Plant Science 10, 287. 30915094 10.3389/fpls.2019.00287 PMC6421314

[CIT0023] Smith ES , Nimchuk ZL. 2023. What a tangled web it weaves: auxin coordination of stem cell maintenance and flower production. Journal of Experimental Botany 74, 6950–6963. doi:10.1093/jxb/erad340 PMC10690728 37661937

[CIT0024] Spaepen S , Vanderleyden J. 2011. Auxin and plant–microbe interactions. Cold Spring Harbor Perspectives in Biology 3, a001438. 21084388 10.1101/cshperspect.a001438 PMC3062209

[CIT0025] Sun Y , Yang B, De Rybel B. 2023. Hormonal control of the molecular networks guiding vascular tissue development in the primary root meristem of Arabidopsis. Journal of Experimental Botany 74, 6964–6974. doi:10.1093/jxb/erad232 PMC7615341 37343122

[CIT0026] Ung KL , Schulz L, Kleine-Vehn J, Pedersen BP, Hammes UZ. 2023. Auxin transport at the endoplasmic reticulum: roles and structural similarity of PIN-FORMED and PIN-LIKES. Journal of Experimental Botany 74, 6893–6903. doi:10.1093/jxb/erad192 37279330

[CIT0027] Van der Does D , Leon-Reyes A, Koornneef A, et al. 2013. Salicylic acid suppresses jasmonic acid signaling downstream of SCFCOI1-JAZ by targeting GCC promoter motifs via transcription factor ORA59. The Plant Cell 25, 744–761. 23435661 10.1105/tpc.112.108548 PMC3608790

[CIT0028] Verma S , Negi NP, Pareek S, Mudgal G, Kumar D. 2022. Auxin response factors in plant adaptation to drought and salinity stress. Physiologia Plantarum 174, e13714. 35560231 10.1111/ppl.13714

[CIT0029] Wakeman A , Bennett T. 2023. Auxins and grass shoot architecture: how the most important hormone makes the most important plants. Journal of Experimental Botany 74, 6975–6988. doi:10.1093/jxb/erad288 PMC10690731 37474124

[CIT0030] Wójcikowska B , Belaidi S, Robert HS. 2023. Game of thrones among AUXIN RESPONSE FACTORs—over 30 years of MONOPTEROS research. Journal of Experimental Botany 74, 6904–6921. doi:10.1093/jxb/erad272 PMC10690734 37450945

